# Impact of αAI-1 Expressed in Genetically Modified Cowpea on *Zabrotes subfasciatus* (Coleoptera: Chrysomelidae) and Its Parasitoid, *Dinarmus basalis* (Hymenoptera: Pteromalidae)

**DOI:** 10.1371/journal.pone.0067785

**Published:** 2013-06-28

**Authors:** Christoph Lüthi, Fernando Álvarez-Alfageme, Jörg Romeis

**Affiliations:** Agroscope Reckenholz-Tänikon Research Station ART, Zürich, Switzerland; French National Institute for Agricultural Research (INRA), France

## Abstract

Genetically modified (GM) cowpea seeds expressing αAI-1, an α-amylase inhibitor from the common bean, have been shown to be immune against several bruchid species. Effective control of such pests by growing GM cowpea could promote the spread of bruchid species that are αAI-1 tolerant. Consequently, the sustainability of bruchid pest control could be increased by combining GM seeds and hymenopteran parasitoids. However, there are concerns that αAI-1 could interfere with the biological control provided by parasitoids. Here, we assessed the impact of GM cowpea seeds expressing αAI-1 on the αAI-1-tolerant bruchid *Zabrotes subfasciatus* and its parasitoid *Dinarmus basalis*. αAI-1 in cowpea seeds did not increase resistance to *Z. subfasciatus* or affect the mortality rate of *Z. subfasciatus* larvae. Parasitism of *Z. subfasciatus* by *D. basalis* and fitness of *D. basalis* offspring were not affected by the presence of αAI-1. Thus, αAI-1-expressing cowpeas and parasitoids should be compatible for the control of bruchid pests.

## Introduction

Cowpea (*Vigna unguiculata*) is the predominant legume crop in West Africa, a region that is responsible for more than 80% of the global cowpea production (FAOSTAT: http://faostat.fao.org). The adaptation of the plant to the local climate, its high nutritional quality, and the storability of the dry seeds make cowpea a staple food for small-scale subsistence farmers. The major pests of stored cowpea seeds and other stored legume seeds are bruchid beetles (Coleoptera: Chrysomelidae: Bruchinae). In the case of cowpea, the two predominant bruchid pests are *Callosobruchus maculatus* and *C. chinensis* [1]. The observation that seeds of the common bean (*Phaseolus vulgaris*) are resistant to several bruchid species, including the two *Callosobruchus* spp., led to the identification of the storage protein αAI-1 as a main resistance factor [2]. αAI-1 is an α-amylase inhibitor and is toxic to bruchids because it inhibits their α-amylases, which are key enzymes in their digestion of long-chain carbohydrates. The gene coding for αAI-1 has been transferred into other legumes, including cowpea, which were subsequently protected against several bruchid species [1], [3]–[10]. Genetically modified (GM) cowpea expressing αAI-1 under the seed-specific promoter of the common bean phytohemagglutinin gene (*dlec2*) is resistant to the two *Callosobruchus* spp. [1], [10], [11]. However, at least two cosmopolitan bruchid species considered as secondary cowpea pests, *Zabrotes subfasciatus* and *Acanthoscelides obtectus* [12], are tolerant to αAI-1 [13], [14] and αAI-1-expressing GM chickpea and cowpea seeds are as susceptible to *A. obtectus* as non-transformed seeds [11]. Progress in management of the predominant *Callosobruchus* spp. by growing αAI-1 GM cowpea might therefore be erased by the spread of secondary bruchid pests or by the development of resistance in the hitherto susceptible species. It has therefore been suggested that bruchid management might be more sustainable if control by αAI-1 GM cowpea was combined with control by hymenopteran parasitoids, which are important natural enemies of bruchids [15]. However, Álvarez-Alfageme et al. [16] demonstrated that under *in vitro* conditions, the α-amylases of several important bruchid parasitoids are as susceptible to αAI-1 as those of the *Callosobruchus* spp. Accordingly, parasitoids attacking bruchid larvae tolerant to αAI-1 and developing in GM seeds might be directly harmed (by host-mediated exposure) or indirectly harmed (by reduced host quality) by the presence of αAI-1 in the seeds. This might lead to a decline in the control provided by the parasitoids and might ultimately promote the spread of secondary bruchid pests.

In this study, we investigated the compatibility of αAI-1 GM cowpea and bruchid parasitoids. The study included three independently transformed cowpea lines expressing αAI-1 and their respective controls, the αAI-1 tolerant bruchid *Z. subfasciatus*, and the αAI-1 susceptible parasitoid *Dinarmus basalis* (Hymenoptera: Pteromalidae). *Dinarmus basalis* is a solitary ectoparasitoid of bruchid larvae and pupae.

## Materials and Methods

### Insects

Our laboratory colony of *Z. subfasciatus* was established with a strain provided by Isabelle Zaugg (University of Fribourg, Switzerland). This strain had originally been collected on wild beans in Mexico.

*Dinarmus basalis* was provided by J.P. Monge (Tours University, France). The parasitoids were reared on *C. chinensis* larvae in chickpea seeds. Before the start of the experiment, the parasitoids were reared on *Z. subfasciatus*-infested cowpea seeds for at least two generations. The insects were maintained and the experiments were conducted in a climate chamber at 24±2°C, 40±5% r.h, and complete darkness.

### Cowpea Seeds

GM cowpea seeds expressing αAI-1 were developed at CSIRO Plant Industry (Australia) and provided together with the corresponding control lines by T.J.V. Higgins (CSIRO Plant Industry, Australia) [10], [17]. Seeds with two different plant backgrounds (cultivars IT86D-1010 and Sasaque) were used. IT86D-1010 was the parent for the GM line TCP 14A and the corresponding null-pair line NTCP 14A. Sasaque was the parent for three independently transformed lines (T170, T239, and T310) and their corresponding null-pair lines (NT170, NT239, and NT310). Experiment 1 (the “bitrophic” experiment) included the parental, the GM, and the null-pair line of cultivar IT86D-1010, the three pairs of GM and null-pair lines of cultivar Sasaque, plus the cowpea type that was used to breed *Z. subfasciatus*, which was purchased from a local supermarket (i.e., 10 lines in total). All lines with both IT86D-1010 and Sasaque background were shown to have a similar seed coat thickness, which is a relevant factor for bruchid resistance [11]. Experiment 2 (the “tritrophic” experiment) included the following pairs of GM and corresponding null-pair lines: TCP 14A and NTCP 14A, T170 and NT170, and T239 and NT 239. The pair T310/NT310 was not included because of limited number of seeds of these two lines.

### Resistance of Cowpea Seeds to *Z. subfasciatus* (Experiment 1)

A bitrophic experiment was conducted with *Z. subfasciatus* and all cowpea lines. Thirty seeds of each cowpea line were placed individually in open plastic containers (2.2×2.2×1.0 cm), and the 300 plastic containers were arranged randomly in a large box (100×50×20 cm). Approximately 2000 newly emerged adult beetles were released into the box. Seeds were inspected daily for 3 days. Infested seeds (i.e., seeds with eggs attached) were removed from the box and kept individually in plastic containers. Seeds without eggs after 3 days were discarded. This resulted in sample sizes of n = 30 for the lines IT86-1010, NTCP 14A, T170, NT 239, and T 310, n = 29 for the lines NT170, T239, and NT310, and n = 28 for line TCP 14A and the breeding variety. Infested seeds were inspected daily, and as soon as the first larva began chewing into the seed, all other larvae on the same seed were removed with a scalpel to avoid interference among multiple larvae developing in a single seed. Seeds were inspected daily for adult emergence until the experiment was terminated after 70 days. For each line, resistance was calculated as percentage of infested seeds without adult emergence. Seeds without adult emergence were dissected, and the stage of the dead bruchid was determined. We determined whether the bruchid failed to penetrate the seed coat; whether the bruchid penetrated the seed coat but died inside the seed in the larval or pupal stage; or whether the bruchid penetrated the seed coat and successfully completed development but failed to emerge from the seed. Because only bruchid larvae feeding on the cotyledons and embryonic axis of a seed are exposed to plant-expressed αAI-1, larval mortality within the different cowpea lines was analyzed.

### Host-mediated Impact of αAI-1 GM Cowpea Seeds on *D. basalis* (Experiment 2)

A tritrophic experiment was conducted with *D. basalis*, *Z. subfasciatus*, and the following three pairs of GM and null-pair cowpea lines: TCP 14A/NTCP 14A, T170/NT170, and T239/NT239. In the initial bitrophic experiment, we observed a strong effect of the seed coat on the development of *Z. subfasciatus* (Figure S1). Preliminary experiments also revealed that the seed coat affects the emergence rate of *D. basalis*. The seed coats were therefore replaced by an artificial coat according to Shade et al. [18] to exclude this factor in the tritrophic experiment. After 50 seeds per line were soaked in distilled water for 60 to 90 min, their coats were removed. The seeds were dried again for 24 h at 35°C and then dipped into an 8% gelatin solution maintained at 55–60°C**.** The seeds were then put on a glass Petri dish on ice for 5–10 min to quickly solidify the gelatin. The seeds were finally kept in the climate chamber under experimental conditions for 3 days before the start of the experiment.

To infest the seeds with *Z. subfasciatus* larvae, all 50 seeds from one line were placed in a plastic container (10.5 cm diameter, 15 cm high), and approximately 300 adult bruchids were released into the container. Preliminary experiments revealed a lower ovipostion on seeds with artificial seed coat, therefore seeds were offered to *Z. subfasciatus* for maximally 6 days in this experiment. After 2, 4, and 6 days, the seeds were inspected, and those with eggs attached were placed individually in a plastic container (2.2×2.2×1.0 cm). Seeds without eggs after 6 days were considered unsuitable for oviposition and were excluded from the experiment. This resulted in sample sizes ranging from n = 28–39 in the different lines (n for each line is given in Table 1). Infested seeds were inspected regularly, and as soon as a single larva entered a seed, additional eggs and larvae were removed with a scalpel. Once the bruchid larvae reached the fourth larval instar, one young (<24 h) and mated *D. basalis* female was introduced into each container (which contained a single seed and host) and was allowed to oviposit for 24 h. Subsequently, seeds were inspected daily, and if and when the adult parasitoid emerged, its developmental time and sex were recorded. Emergence rate was calculated as the percentage of seeds from which an adult parasitoid emerged. Filial generation 1 (F_1_) males were immediately frozen, and their individual dry weights were measured with a MX5 microbalance (Mettler Toledo, Greifensee, Switzerland) after they had been dried at 60°C for 72 h. Each newly emerged F_1_ female and a single male from the rearing colony were introduced into a plastic container (6 cm diameter, 10 cm high) containing 100 cowpea seeds from the variety that was used to maintain *Z. subfasciatus*. All seeds came from a single pool of seeds that had been exposed to *Z. subfasciatus* over a period of 14 days; this was done to provide suitable hosts for the entire oviposition period of *D. basalis*. For each F_1_ female, realized fecundity (i.e., the total number of offspring) and the offspring sex ratio were recorded.

**Table 1:** Performance of *Dinarmus basalis* on GM or non-GM cowpea lines (experiment 2).

	Females P		Males F_1_		Females F_1_		
Cowpea line^a^	Hosts provided (n)/emergence rate (%)^b^	Sex ratio (f:m)^b^	Developmental time (d)^c^	Dry weight (µg)^c^	Developmental time (d)^c^	Realized fecundity (n)^c^	Mean offspring sex ratio (f:m)^c^
NTCP14A	39/89.7	27∶8	18.5±0.5	281.6±16.1	21.7±0.2	20.6±1.2	0.68±0.052
TCP14A*	36/94.4	23∶11	18.7±0.4	267.2±9.7	21.8±0.3	19.6±1.2	0.66±0.042
	p = 0.68	p = 0.43	p = 0.72 (*t* = −0.37)	p = 0.43 (*t* = 0.81)	p = 0.84 (*t* = −0.20)	p = 0.58 (*t* = 0.56)	p = 0.61 (*t* = 0.51)
NT170	36/94.1	23∶9	19.6±0.4	261.6±11.7	21.6±0.2	19.7±1.3	0.69±0.067
T170*	37/91.7	19∶15	18.8±0.4	251.5±15.3	21.4±0.2	20.7±1.4	0.53±0.069
	p = 0.71	p = 0.21	p = 0.20 (*t* = 1.32)	p = 0.65 (*t* = 0.46)	p = 0.67 (*t* = 0.42)	p = 0.57 (*t* = −0.57)	p = 0.13 (*t* = 1.55)
NT239	28/89.3	17∶8	20.0±0.6	262.9±16.1	21.9±0.4	16.9±1.4	0.64±0.043
T239*	32/90.6	18∶11	19.0±0.4	262.5±7.1	21.9±0.3	18.8±1.5	0.61±0.081
	p = 1	p = 0.78	p = 0.15 (*t* = 1.52)	p = 0.98 (*t* = 0.02)	p = 0.99 (*t* = −0.01)	p = 0.36 (*t* = −0.92)	p = 0.95 (*t* = 0.07)

Performance was assessed as emergence rate and offspring sex-ratio of *D. basalis* parental generation females (P) as well as by the developmental time and dry weight of males and by the developmental time, realized fecundity, and offspring sex ratio of females of the first filial generation (F_1_). P females were provided a single *Zabrotes subfasciatus* larval host developing in a αAI-1 GM or corresponding null-pair control line seed. F_1_ females were provided *Z. subfasciatus* larvae developing in cowpeas of the breeding variety *ad libitum*. Means are presented with standard error.

a GM lines are indicated with an asterisk.

b Values are compared pairwise using Fisher’s exact test.

c Values are compared pairwise using Student’s *t* test.

### Data Analyses

All data were analyzed using the software R (version 2.14.1). In the bitrophic experiment, differences in resistance and larval mortality between the GM and the corresponding null-pair lines were analyzed pairwise using Fisher’s exact test. For the lines based on IT86D-1010, three pairwise comparisons between the parental, GM, and null-pair line were conducted, and the α-level adjusted according to the Bonferroni method, resulting in α = 0.017. In the tritrophic experiment, differences in emergence rate and offspring sex ratio of *D. basalis* females were analyzed pairwise using Fisher’s exact test. Developmental time, dry weight, realized fecundity, and offspring sex ratio of F_1_ individuals were compared pairwise using the parametric Student’s *t* test because the data were normally distributed and the variances were homogenous. Data for the offspring sex ratio of F_1_ females were arcsine transformed prior to the analysis in order to meet the assumptions of the Student’s *t* test.

## Results

### Resistance of Cowpea Seeds to *Z. subfasciatus* (Experiment 1)

**Figure 1:**
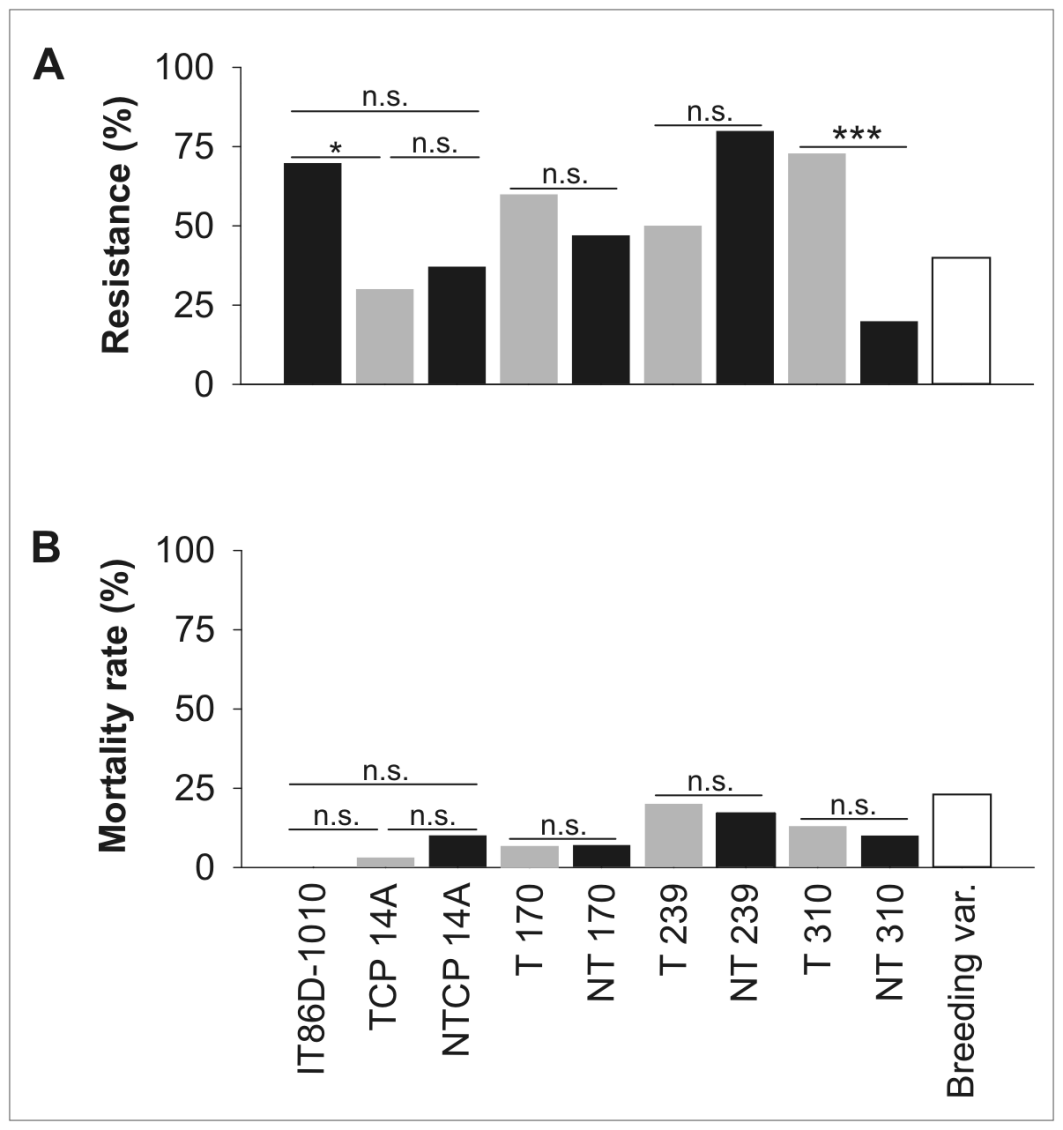
Effects of GM and non-GM cowpea lines on the bruchid pest *Zabrotes subfasciatus* (experiment 1). (A) Resistance (percentage of infested seeds from which no adult beetle emerged) and (B) within-seed larval mortality of *Zabrotes subfasciatus* in different cowpea lines (experiment 1). Comparisons were made among the three IT86D lines (IT86D-1010: parental line; TCP14A: GM line; NTCP14A: null-pair line) and between the transformed (T) and the respective non-transformed (NT) Sasaque lines 170, 239, and 310 using Fisher’s exact test (*p<0.05, ***p<0.01, n.s. = not significant; for the IT86D lines, the α level was adjusted for three pairwise comparisons using the Bonferroni method, resulting in α = 0.017). Grey bars indicate transformed lines and black bars indicate nontransformed lines. “Breeding var.” refers to the variety that was used to breed *Z. subfasciatus*.

The resistance of the cowpea lines to *Z. subfasciatus* is presented in Fig. 1A. The parental IT86D-1010 line was significantly more resistant than the GM line TCP14A (p = 0.004) and was borderline more resistant than the null-pair line NTCP14A (p = 0.019); the difference between the null-pair line NTCP14A and the GM line TCP14A was not significant. Resistance did not differ between the GM and null-pair lines of Sasaque 170 or 239, but resistance was greater for GM line 310 than for its control line (p<0.001). In all lines except the breeding variety (the line used to maintain *Z. subfasciatus*), most mortality was caused by the failure of larvae to chew through the seed coat (Figure S1).

Results for the within-seed larval mortality of *Z. subfasciatus* in the different cowpea lines are presented in Fig. 1B. Within-seed larval mortality was generally low in all lines and was absent in the parental IT86D-1010 line. None of the pairwise comparisons revealed significant differences in mortality rate.

### Host-mediated Impact of αAI-1 GM Cowpea Seeds on *D.* *basalis*

Results of the tritrophic experiment are presented in Table 1. The number of seeds per line containing a suitable host, i.e., the sample size per line, ranged from 28 to 39. Emergence rate ranged from 89.3 to 94.4% and did not significantly differ between GM lines and their respective controls. The sex ratio in F_1_ was female-biased in all lines, ranging from 0.56 to 0.77 but did not significantly differ between GM lines and their respective controls.

In all cases, developmental time of *D. basalis* larvae was significantly longer for females than for males (Student’s *t* test, *t* = 11.7, p<0.01) and was therefore separately analysed for the two sexes. The comparison for females and for males was not significant in any of the three pairwise comparisons.

The impact of the GM vs. non-GM lines on the F_1_ adults was analyzed by comparing the dry weight of males and the realized fecundity of females. Male adult dry weight did not significantly differ between the GM lines and their respective controls. Realized fecundity or the offspring sex ratio of the females also did not significantly differ between the GM lines and their respective controls.

## Discussion

*Zabrotes subfasciatus* is known to be tolerant to αAI-1 [13], [14] and should therefore not be affected by the presence of this inhibitor in the GM cowpea seeds. Indeed, none of the GM lines expressing αAI-1 was immune to this bruchid, and mortality of larvae, the stage that is exposed to αAI-1, was low in all lines. Nevertheless, resistance did differ in two of the comparisons of GM and null-pair lines. A similar result has been found for the other αAI-1 tolerant bruchid, *A. obtectus* [11]. The low mortality during the larval stage inside the seeds indicates that factors other than αAI-1 are responsible for the observed differences in seed resistance. It has been argued that the seed coat of non-host legumes can contribute to the resistance against bruchids [19], and in the current study, the failure to chew through the seed coat accounted for the highest larval mortality in experiment 1. Cowpea is not a primary host of *Z. subfasciatus*, and the cowpea seed coat contains resistance factors such as tannins and α-amylase inhibitors [20]. The concentrations of resistance factors in the seed coat might have been affected by tissue culture, which was used in the generation of GM plants, because tissue culture can lead to phenotypic changes, so called somaclonal variation [21], [22]. Because seed coat thickness was shown to be similar in all IT86D and Sasaque lines included in this study [11], we infer that this resistance factor was not responsible for the observed differences in resistance to *Z. subfasciatus*.

The similar larval mortality observed in all lines investigated in the bitrophic experiment and the use of an identical artificial seed coat in the tritrophic experiment indicate that the quality of *Z. subfasciatus* larvae as hosts for *D. basalis* in the tritrophic experiment did not differ between the GM and their corresponding null-pair lines except for the presence or absence, respectively, of αAI-1. Thus host-quality-mediated (indirect) effects on bruchid parasitoids should be negligible.

Females of hymenopteran parasitoids are able to evaluate host quality and adjust their egg-laying behavior accordingly [23]. Female *D. basalis* are synovigenic, i.e., they rely on nutrient intake to develop mature eggs sequentially, and they therefore feed on host hemolymph. Although oviposition and host feeding is typically non-concurrent in hymenopteran parasitoids [24], [25], it is not known whether this also true for *D. basalis*. We therefore have to assume that upon finding hosts, *D. basalis* females have to decide whether (i) to feed, (ii) to oviposit, or (iii) both and, when ovipositing, whether to deposit an egg that produces a male or a female offspring. An influence of host quality on sex ratio has been documented for *D. basalis*, with lower host quality resulting in a male-biased offspring ratio [26], [27]. If host quality does also influence the decision whether to oviposit or to feed on the host, this would result in a reduced offspring production from lower quality hosts. The ingestion of αAI-1 by the host might influence host quality and hence the behavior of *D. basalis* females encountering it. In the current study, however, the emergence rate and the offspring sex ratio did not differ between GM lines and their controls. It seems that hosts developing in αAI-1 GM and null-pair seeds were of similar quality and did not affect the parasitic behavior of *D. basalis*. Additionally, a negative impact on host-feeding females is unlikely because direct feeding studies with αAI-1 have shown that *Anisopteromalus calandrae*, a parasitoid that is closely related with *D. basalis*, is relatively tolerant of αAI-1 [16]. Taken together, the effect of αAI-1 GM seeds on oviposition and on host-feeding females appears to be negligible.

Parasitoid larvae depend greatly on the host quality because the host is their only source of nutrition. This is particularly true for idiobiont species like *D. basalis*, where the host does not grow after parasitism and the quality and quantity of nutrients available for the developing parasitoid larva are therefore fixed. Although the *D. basalis* larvae were likely exposed to αAI-1 when consuming a host in a GM seed, male and female larval developmental time, which is one of the most important fitness parameters of parasitoids [28], did not differ between GM lines and their control lines. This indicates that the consumption of αAI-1 by *Z. subfasciatus* did not influence parasitoid development. Similarly, parasitoid larvae that developed into adults on hosts in GM seeds did not seem to suffer from reduced fitness in terms of adult male dry weight, male and female developmental times, realized fecundity, and offspring sex ratio. Lacoume et al. [27] showed that host size, which is an important component of host quality, affects the body size of *D. basalis* males and that smaller males suffered from mating disadvantages compared to larger males. The absence of a significant difference in dry weight and developmental time is strong evidence that males are not affected by αAI-1 in the GM seeds. In the case of the F_1_ females, developmental time, realized fecundity, and offspring sex ratio were not significantly different when the females had developed on hosts in GM or control seeds. Because F_1_ females were provided hosts in similar quantity and of similar quality for oviposition, we conclude that the fitness of the F_1_ females was most likely not affected by the presence of αAI-1 in the GM seeds. However, given the set-up of our experimental system, we cannot totally rule out the alternative explanation that female oviposition rates differed between the corresponding cowpea lines, what was then compensated by differences in larval mortality, resulting in similar offspring rates.

Although host-mediated impacts of several plant metabolites on hymenopteran parasitoids have been described (e.g., reviewed in [29]), we did not observe significant effects of αAI-1 expressed in the GM seeds on *D. basalis*. Yet, the level of exposure of *D. basalis* to host-mediated αAI-1 remains unclear. Because α-amylases of both larvae and females of *D. basalis* are strongly inhibited by αAI-1 [16], the exposure was probably too low to cause a detectable effect. Additionally, *in vitro* assays showed that midgut extracts of *Z. subfasciatus* larvae rapidly digest αAI-1 and thus inactivate the inhibitor [13], [14]. If such inactivation of αAI-1 also occurs *in vivo*, it could at least partially explain why GM cowpea, expressing αAI-1 and infested with *Z. subfasciatus*, did not negatively affect the parasitoid. Finally, *D. basalis* is a common parasitoid of *Z. subfasciatus* and *A. obtectus*, which are both pests of the common bean [12]. Thus, *D. basalis* might have encountered αAI-1, which is naturally present in common bean seeds, in its evolutionary history and found ways to cope with it. In this context, a second plant resistance factor from the common bean, arcelin, did not interfere with the control of *A. obtectus* by *D. basalis* [30]. Furthermore, the combination of plant resistance factors and *D. basalis* has been found to improve the control of *A. obtectus* and *Z. subfasciatus* [31], [32]. Therefore, the evidence indicates that αAI-1 GM cowpeas are not only compatible with biological control services provided by *D. basalis*, but that their combination could provide an improved and sustainable management of bruchid pests.

## Supporting Information

Figure S1 **Mortality rates of** ***Zabrotes subfasciatus*** **developing in the three IT86D lines (IT86D: parental line; TCP14A: GM line; NTCP14A: null-pair line); in the transformed (T) and respective non-transformed (NT) Sasaque lines 170, 239, and 310; and in the breeding variety (experiment 1).** Stacked bars represent total mortality, and single stacks indicate mortality rates for each respective stage (“entering”: larva failed to drill into seed; “larva”, “pupa”: beetle died in the respective stage inside the seed; “adult”: adult beetle failed to emerge from the seed). “Breeding var.” refers to the variety that was used to breed *Z. subfasciatus*.
(TIF)
